# Supplementary figures and images for: Heterogeneity of Response to Iron-Based Metallodrugs in Glioblastoma Is Associated with Differences in Chemical Structures and Driven by FAS Expression Dynamics and Transcriptomic Subtypes
Source: Int J Mol Sci. 2021 Sep 27;22(19):10404. doi: 10.3390/ijms221910404 (PMC8508975; doi:10.3390/ijms221910404)

## Slide 1
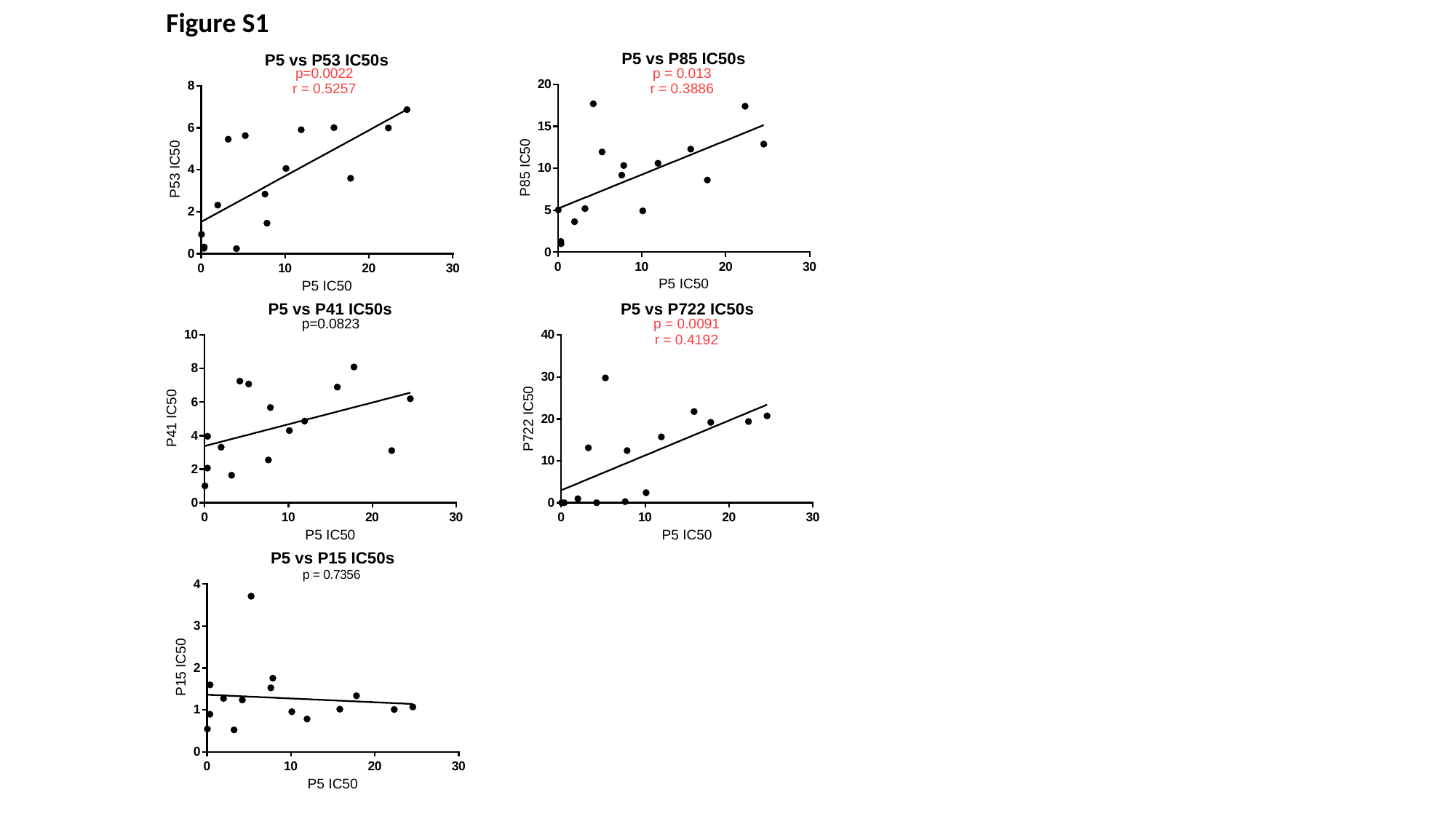

Figure S1

## Slide 2
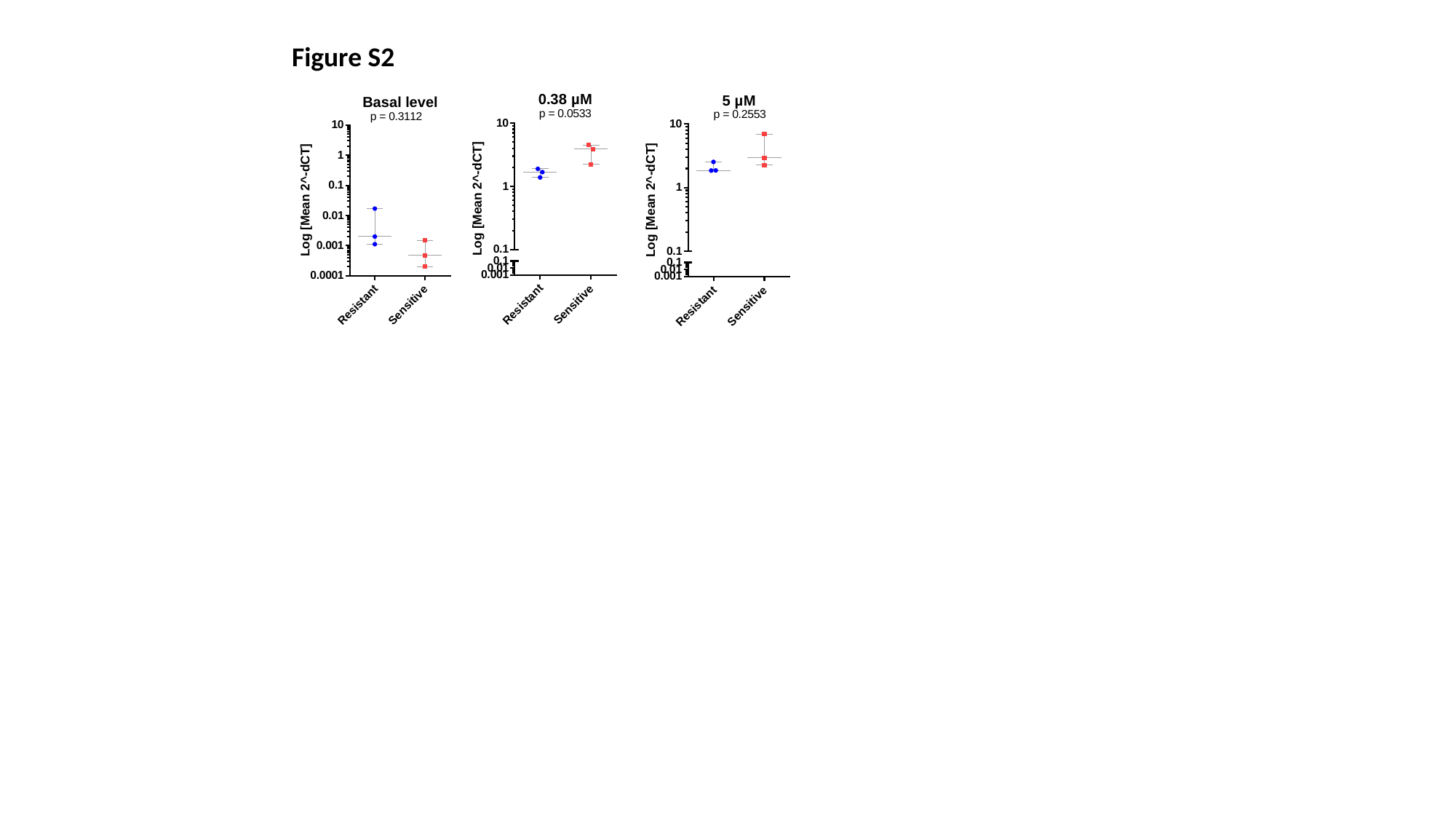

Figure S2

## Slide 3
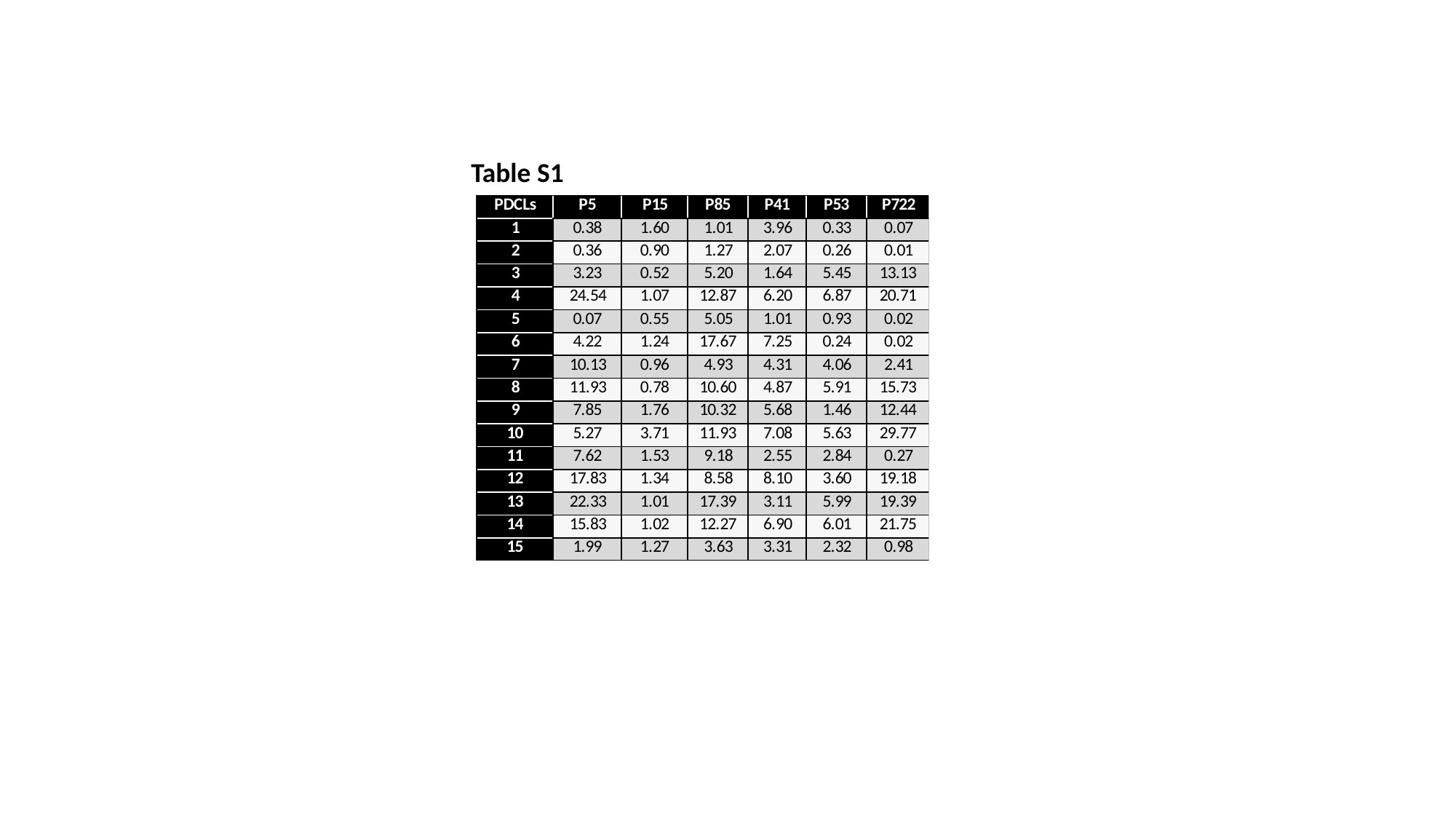

Table S1

## Slide 4
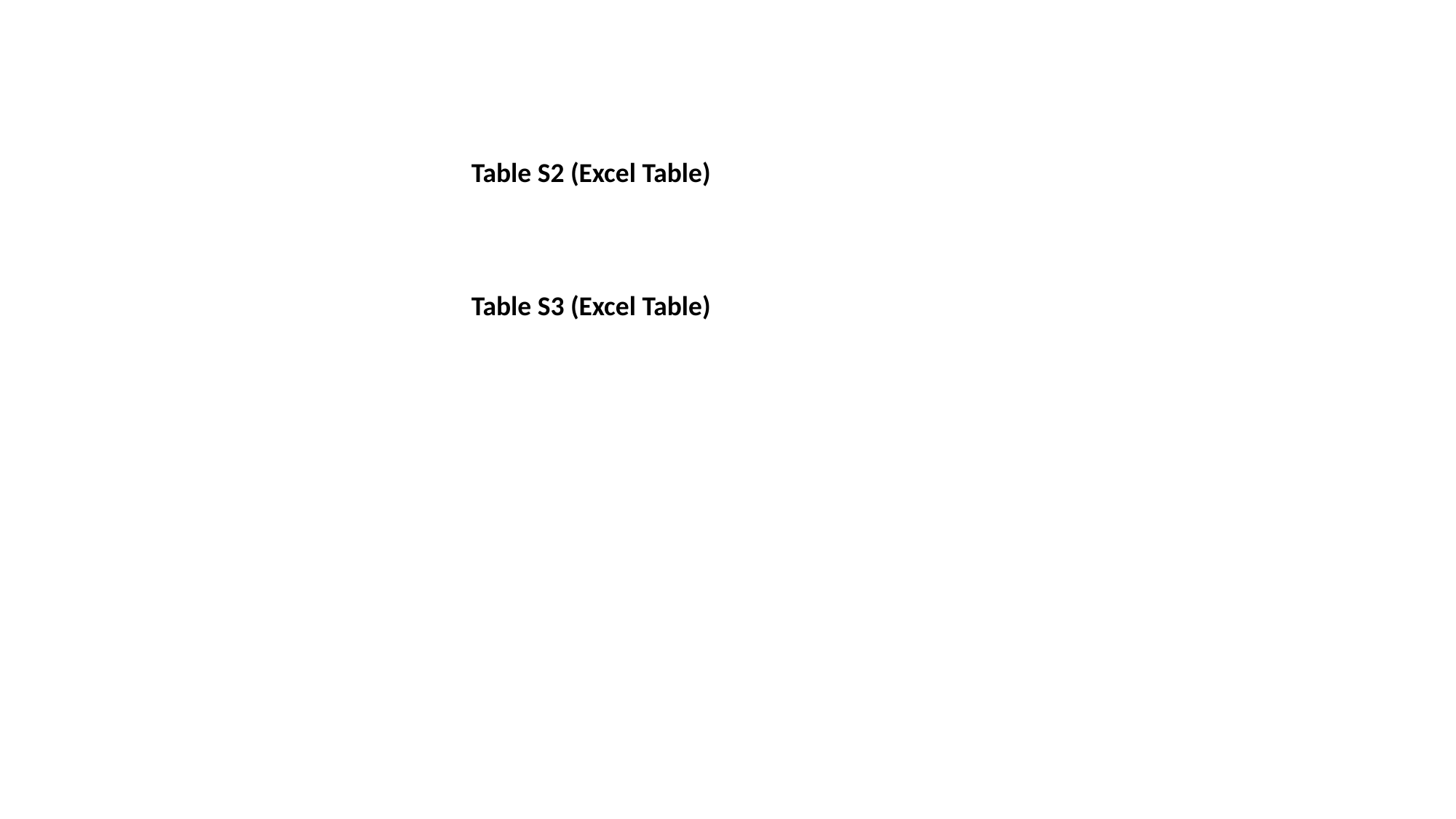

Table S2 (Excel Table)
Table S3 (Excel Table)

Supplement: Supplementary file 1 [file ijms-22-10404-s001.zip › Ferrocene manuscript - supplementary figures and table.pptx]
